# Evaluating programmed death‐ligand 1 (PD‐L1) in head and neck squamous cell carcinoma: concordance between the 22C3 PharmDx assay and the SP263 assay on whole sections from a multicentre study

**DOI:** 10.1111/his.14562

**Published:** 2021-11-11

**Authors:** Bruna Cerbelli, Ilaria Girolami, Albino Eccher, Leopoldo Costarelli, Silvia Taccogna, Renzo Scialpi, Maria Benevolo, Teresa Lucante, Piero Luigi Alò, Francesca Stella, Maria Gemma Pignataro, Guido Fadda, Giuseppe Perrone, Giulia D’Amati, Maurizio Martini

**Affiliations:** ^1^ Department of Medico‐Surgical Sciences and Biotechnologies Sapienza University of Rome Rome Italy; ^2^ Division of Pathology Central Hospital Bolzano Bolzano Italy; ^3^ Department of Pathology and Diagnostics University and Hospital Trust of Verona Verona Italy; ^4^ Department of Pathology S. Giovanni‐Addolorata Hospital Rome Italy; ^5^ Anatomical Pathology Unit Regina Apostolorum Hospital Albano Laziale Italy; ^6^ Unità Operativa complessa di Anatomia Patologica Ospedale Sandro Pertini Rome Italy; ^7^ IRCCS Regina Elena National Cancer Institute Rome Italy; ^8^ Unità Operativa Complessa Anatomia Patologica Ospedale San Giovanni Calibita Fatebenefratelli Rome Italy; ^9^ Unità Operativa Complessa Anatomia Patologica Ospedale Fabrizio Spaziani Frosinone Italy; ^10^ Unità Operativa Complessa Anatomia Patologica Ospedale San Camillo‐Forlanini Rome Italy; ^11^ Department of Radiological, Oncological and Pathological Sciences ‘Sapienza’ University of Rome Rome Italy; ^12^ Dipartimento di Patologia Umana dell’adulto e dell’età evolutiva Gaetano Barresi Messina Italy; ^13^ Research Unit of Pathology Campus Bio‐Medico University Rome Italy; ^14^ Department of Health Science and Public Health Division of Pathology Università Cattolica del Sacro Cuore Rome Italy

**Keywords:** 22C3 assay, head and neck squamous carcinoma, PD‐L1, SP263 assay

## Abstract

**Aims:**

The introduction of immunotherapy for patients with head and neck squamous cell carcinoma (HNSCC) raises the need for harmonisation between different types of antibody and immunohistochemistry platform for evaluating the expression of PD‐L1 by use of the combined positive score (CPS) in this tumour. The aim of this study was to compare the expression of PD‐L1 as determined with the CPS and two widely used assays (the 22C3 PharmDx assay and the SP263 assay) in a cohort of HNSCCs.

**Methods and results:**

We analysed 43 whole sections of HNSCC with two different anti‐PD‐L1 antibodies, 22C3 and SP263. The results, expressed as the CPS, were evaluated by 10 trained pathologists and statistical analyses were performed. We found a very similar results for PD‐L1 expression between the 22C3 PharmDx assay and the SP263 assay in our cohort, and a strong and significant correlation between the two assays for all specimens (*P* < 0.0001). The interobserver reliability among pathologists for the continuous scores of CPS with the intraclass correlation coefficient and the correlation between the two assays were both good. Moreover, the rate of agreement between assays was high at all cut‐offs and was best for the most relevant cut‐off of CPS ≥ 1, and the kappa values were always in the range of almost perfect.

**Conclusions:**

Two different assays (the 22C3 PharmDx assay and SP263 assay) for PD‐L1 in HNSCC showed high agreement. These data suggest that these two assays are interchangeable in the selection of patients with HNSCC for immunotherapy.

## Graphical abstract

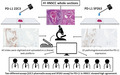

## Introduction

Head and neck cancer represents the sixth most common type of cancer worldwide, with a prevalence of 6% translating into 650 000 new cases per year.^1^ Almost 90% of head and neck cancers are of the squamous cell type [head and neck squamous cell carcinoma (HNSCC)]. At present, the standard of care for locally advanced HNSCC is based on primary resection followed by radiochemotherapy treatment.^2^, ^3^ Despite this intensive multimodality therapy, survival is poor, owing to the high frequency of disease recurrence.^4^, ^5^ A turning point in the therapy of HNSCC was the introduction of immunotherapy targeting the programmed death‐1 (PD‐1)/programmed death‐ligand‐1 (PD‐L1) axis, resulting in an improvement in overall survival.^6^, ^7^, ^8^ Recent trials investigating the efficacy of this first‐line immune checkpoint inhibition for recurrent and/or metastatic HNSCC showed that PD‐L1 expression is associated with an increased objective response rate in patients with a combined positive score (CPS) of ≥1, with a better response being seen when the CPS was ≥20.^9^, ^10^ These last studies allowed the Federal Drug Administration (FDA; 2019) to approve pembrolizumab in combination with platinum and fluorouracil, regardless of PD‐L1 status, for the treatment of recurrent and/or metastatic HNSCC, and for monotherapy in patients with CPS ≥ 1 evaluated with an FDA‐approved test.^11^ The FDA also expanded the intended use for the PD‐L1 immunohistochemistry (IHC) 22C3 PharmDx assay to include its use as a companion diagnostic device for selecting patients with HNSCC for treatment with pembrolizumab. In 2020, the European Medicines Agency (EMA) (https://www.ema.europa.eu/en/documents/variation‐report/keytruda‐h‐c‐3820‐ii‐0065‐epar‐assessment‐report‐variation_en.pdf) and the UK’s National Institute for Health and Care Excellence (NICE), with their guideline documents, approved pembrolizumab, both as monotherapy and in combination with chemotherapy, as a first‐line treatment for metastatic or unresectable recurrent HNSCC in patients whose tumours express PD‐L1 with CPS ≥ 1, regardless of the test (antibody and IHC platform) used.^12^, ^13^ Unfortunately, several antibody clones and platforms have been used for the evaluation of PD‐L1 expression, making comparison among these difficult, especially as the literature data regarding HNSCC are poor.

In this scenario, the availability of a reproducible and robust immunohistochemical method for determining the CPS is of major importance, and the interchangeability of different assays requires appropriate validation and harmonising studies.^14^, ^15^ Another important point to consider when PD‐L1 expression is assessed, especially with use of the CPS, is concordance among pathologists. Indeed, the CPS is more complex and perhaps less intuitive than the tumour proportion score, as it requires specific counting of tumour and immune cells in order to calculate the score. Not surprisingly, training in this regard has been shown to be important.^16^ To date, only a few studies have focused specifically on CPS evaluation in HNSCC. Both are based on the analysis of tissue microarrays (TMAs) and highlight variable degrees of agreement between the different assays, the reference standard, and laboratory‐developed tests (LDTs).^17^, ^18^ These results raise concerns about the interchangeability of the available tests. Moreover, the evaluation of whole sections, which represent the ‘real‐life’ setting, instead of TMAs could further affect the degree of concordance between the different diagnostic tests, and, perhaps, the interobserver variability.

The aim of our study was to compare the diagnostic performances of two most widely used assays, i.e. the 22C3 PharmDx assay performed on the Agilent Autostainer Link 48 versus the SP263 assay performed on the Ventana Benchmark XT staining system, at clinically relevant cut‐offs (≥1 and ≥20) on whole sections from a multicentre cohort of HNSCCs.

## Materials and methods

### Sample Collection And Evaluation

The present study was a multicentre observational retrospective study of 43 patients with metastatic or unresectable recurrent HNSCC who underwent biopsy or surgical resection (27 biopsies and 16 surgical specimens). A maximum of five samples was collected from each of the 10 participating regional hospitals. Patients undergoing neoadjuvant chemotherapy and/or radiotherapy were excluded from this study. Fixation of the specimens was performed with 10% buffered formalin, with exposure from 12 to 48 h. Then, biopsies or surgically resected HNSCC samples were paraffin‐embedded and, for each case, a representative haematoxylin and eosin (H&E)‐stained slide was obtained. Human papillomavirus status in oropharyngeal squamous cell carcinoma (SCC) and in metastases was assessed with the CINtec p16 Histology assay (Roche, Milan, Italy), with strong and diffuse nuclear and cytoplasmic staining in at least 70% of cells being used as the cut‐off for positivity. All patient data were collected anonymously, and written informed consent, as part of the routine diagnosis and treatment procedures, was obtained from patients or their guardians according to the Declaration of Helsinki. The study adhered to Good Clinical Practice guidelines.^19^

### PD‐L1 IHC And Interpretation

PD‐L1 immunohistochemistry was performed on each specimen (3‐μm‐thick consecutive sections) with two anti‐PD‐L1 antibodies, clone 22C3 and SP263, according to the manufacturer’s instructions. Briefly, we used the 22C3 PharmDx assay (mouse monoclonal primary anti‐PD‐L1 antibody, prediluted, clone 22C3; Dako, Carpinteria, CA, USA) on the Autostainer Link 48 with the EnVision DAB Detection System (Agilent Technologies, Santa Clara, CA, USA), and the SP263 assay (rabbit monoclonal primary anti‐PD‐L1 antibody, prediluted; Ventana Medical Systems, Tucson, AZ, USA) on the Benchmark XT staining system and the OptiView Universal DAB Detection Kit (Ventana Medical Systems). Immunohistochemical analysis for both anti‐PD‐L1 antibodies was centralised and performed at the Molecular Pathology Laboratory of Università Cattolica del Sacro Cuore. PD‐L1 control slides from the 22C3 PharmDx assay (containing sections of two pelleted, formalin‐fixed paraffin‐embedded cell lines: NCI‐H226 with moderate PD‐L1 expression, and MCF‐7 without PD‐L1 expression) were used as positive and negative controls for both antibodies (22C3 and SP263). We also used placental, tonsil and vermiform appendix tissues as positive controls. All slides (H&E and PD‐L1 stains) were digitised with an Aperio CS2 (Leica Biosystems, Milan, Italy) at ×40, uploaded on a shared web platform provided by Nikon (Nikon Europe, Milan, Italy), and viewed with ndp.view2 software by head and neck pathologists specifically trained and certified in CPS assessment from each participating centre. The evaluation was performed on whole slides, and the CPS was determined as the number of PD‐L1‐positive tumour cells, lymphocytes and macrophages divided by the total number of viable tumour cells, multiplied by 100. Any perceptible and convincing partial or complete linear membranous staining of viable tumour cells that was perceived as distinct from cytoplasmic staining was considered to be positive PD‐L1 staining and was included in the scoring. Likewise, any membranous and/or cytoplasmic staining of mononuclear inflammatory cells within tumour nests and/or adjacent supporting stroma was considered to be positive PD‐L1 staining and was included in the CPS numerator. Neutrophils, eosinophils, plasma cells and inflammatory cells associated with *in‐situ* components, benign structures or ulcers were excluded from the CPS. The CPS cut‐offs of ≥1 and ≥20 were investigated. Each countable section contained at least 100 viable HNSCC cells. All pathologists (*n* = 10) received appropriate training for CPS evaluation in HNSCC (certified pathologists) and were blinded to clinical information and the evaluation results of other pathologists.

### Statistical Analysis

The interobserver reliability of pathologists regarding the CPS was determined by calculating the intraclass correlation coefficient (ICC) for each assay. Correlation among the continuous values of CPS between the two assays was assessed with the ICC based on a single‐rating, absolute agreement, two‐way mixed model. The level of agreement on PD‐L1 expression between assays was determined by the use of Cohen’s kappa with confidence intervals (CIs) for each cut‐off after stratification of cases among the relevant cut‐offs. Overall percentage agreement (OPA) with 95% CIs at each cut‐off value (≥1, ≥20, and for the three categories together), positive percentage agreement (PPA) and negative percentage agreement (NPA) were calculated. Statistical analyses were performed with Microsoft Excel 2013, ibm spss statistics for Windows, Version 25.0 (IBM, Armonk, NY, USA) and r software version 4.0.0 (R Foundation for Statistical Computing, Vienna, Austria).

## Results

### Patient Characteristics And PD‐L1 Staining With Use of The 22C3 PHARMDx ASSAY And The SP263 Assay

**Figure 1:**
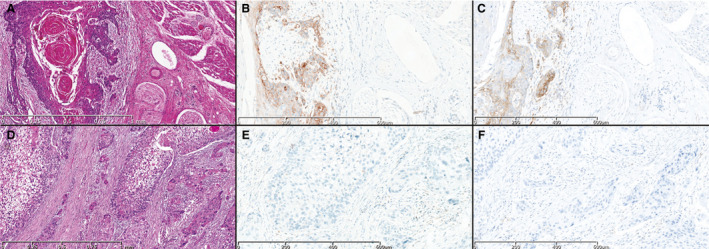
Two head and neck squamous cell carcinoma samples (**A, D,** haematoxylin and eosin) analysed with the 22C3 PharmDx assay (**B, E**) and the SP263 assay (**C, F**). Programmed death‐ligand 1 expression as determined with the 22C3 and SP263 antibodies shows similar combined positive score (CPS) values in the two cases (**A**–**C,** CPS ≥ 20; **D**–**F,** CPS < 20).

**Figure 2:**
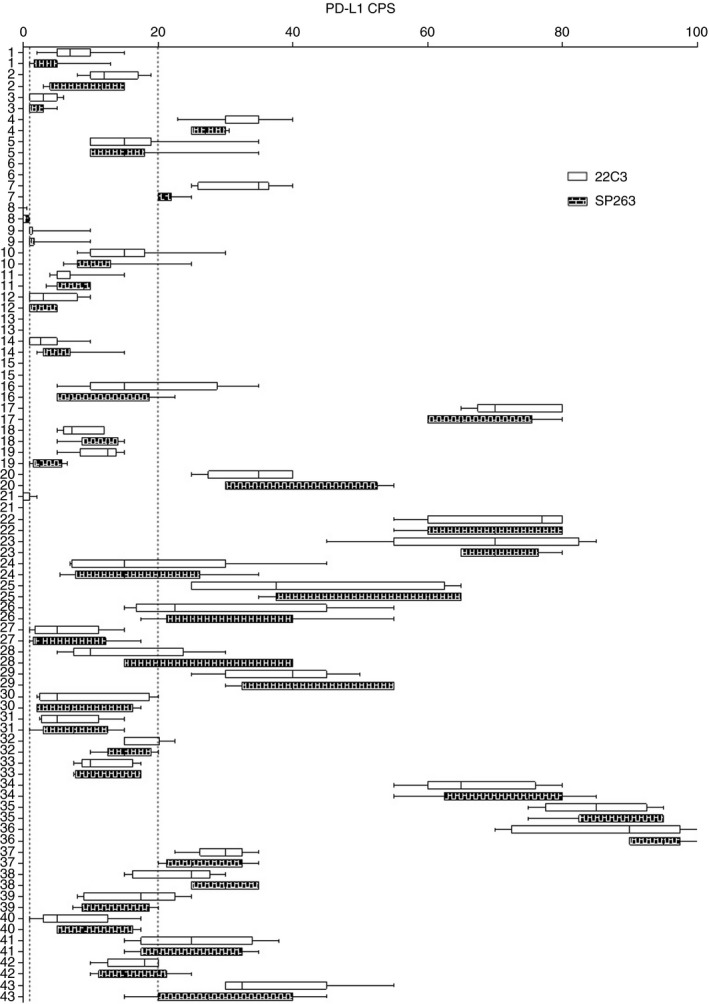
The boxplot of the distribution of programmed death‐ligand 1 combined positive score (CPS) values (clear box for 22C3 and coloured box for SP263) for all samples. The smallest value and the largest value are at the ends of the ‘whiskers’, and the interquartile range is the box. The two dotted lines in the plot indicate CPS cut‐offs of 1 (near the *y*‐axis) and 20.

**Figure 3:**
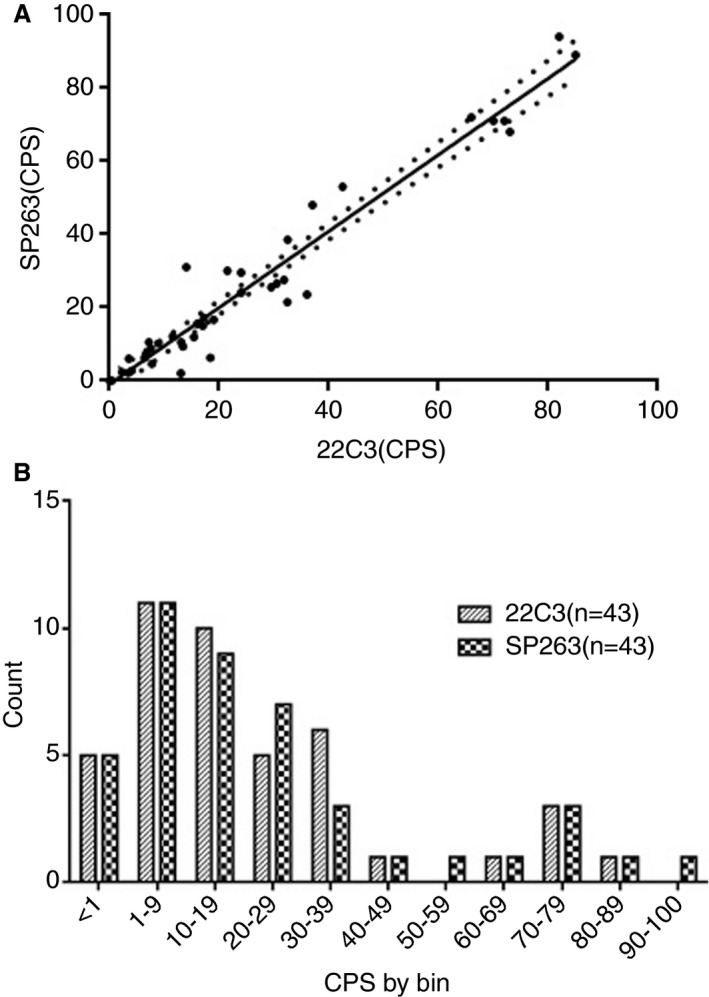
**A,** The direct and significant correlation between the combined positive score (CPS) evaluated with the 22C3 antibody and the SP263 antibody (Spearman *r* = 0.945; *P* < 0.0001). **B,** The distribution of programmed death‐ligand 1 expression as determined with the the 22C3 PharmDx assay kit and the SP263 assay for the CPS.

The sample analysed included 27 (62.8%) biopsies and 16 (37.2%) surgical specimens of HNSCCs collected from 2020 to 2021, giving a total of 43 digitised cases. The main clinicopathological characteristics of our cohort are shown in Table 1. The mean age at the time of diagnosis was 61 years, and 72% of the patients were male. Thirty‐two patients (74.4%) had metastatic disease and 11 (25.6%) had unresectable/recurrent neoplasia. Seventeen of 43 patients (39.5%) had SCC located in the oropharynx, 13 of 43 (30.2%) had SCC located in the hypopharynx, eight of 43 (18.6%) had SCC located in the larynx, and five of 43 (11.7%) had metastatic localisations. Seven of 22 patients (31.8%, including 17 cases of oropharyngeal SCC and five cases of SCC metastasis) showed p16 expression and 15 of 22 did not (68.2%). PD‐L1 IHC was performed with the 22C3 PharmDx assay and the SP263 assay, and the CPS was calculated for each sample. Representative IHC images and boxplots of CPS values of PD‐L1, determined with the 22C3 PharmDx assay and the SP263 assay, showed similar staining patterns and distributions for the same samples (Figures 1 and 2). When we applied the cut‐off of ≥1, 38 samples (88.4%) had positive CPS values with both the 22C3 PharmDx assay and the SP263 assay. CPS values between 1 and 20 were found, respectively, in 21 of 43 (48.8%) samples with the 22C3 PharmDx assay and in 20 of 43 (46.5%) samples with the SP263 assay. CPS values of ≥20 were found in 17 of 43 (39.5%) samples with the 22C3 PharmDx assay and in 18 of 43 (41.9%) samples with the SP263 assay. Five samples (11.7%) had CPS < 1 with both assays. We found a strong and significant correlation between the two assays for all specimens when we compared the CPS values determined with the 22C3 PharmDx assay and those determined with the SP263 assay (Spearman *r* = 0.945; *P* < 0.0001; Figure 3A). The distribution of CPS values is shown in Figure 3B. We found a very similar distribution for the two assays, although the number of samples with CPS ≥ 20 were slightly higher in the group analysed by SP263 assay than the group analysed by 22C3 PharmDx assay.

**Table 1:** Patient characteristics (*N* = 43)

Characteristics	Value
Age (years), mean ± SD	61 ± 8.7
Sex, *n* (%)	
Male	31 (72.1)
Female	12 (27.9)
Stage, *n* (%)	
Metastatic	32 (74.4)
Unresectable recurrent	11 (25.6)
Tumour location, *n* (%)	
Oropharynx	13 (30.2)
Hypopharynx	17 (39.5)
Larynx	8 (18.6)
Metastatic sites	5 (11.7)
HPV status (p16), *n* (%)	
Positive	7 (31.8)
Negative	15 (68.2)
PD‐L1 expression, 22C3, *n* (%)	
<1	5 (11.7)
1 to <20	21 (48.8)
≥20	17 (39.5)
PD‐L1 expression, SP263, *n* (%)	
<1	5 (11.7)
1 to <20	20 (46.5)
≥20	18 (41.8)

HPV, human papillomavirus; PD‐L1, programmed death‐ligand 1; SD, standard deviation.

### Interobserver Agreement

To evaluate the interobserver agreement on PD‐L1 interpretation, we analysed the results of CPS evaluation by 10 pathologists on the 43 HNSCC samples (Table 2). Interobserver reliability among pathologists for the continuous scores of the CPS with the ICC were 0.834 (CI 0.758–0.896) and 0.868 (CI 0.803–0.918) for the 22C3 PharmDx assay and for the SP263 assay, respectively. The correlation between the two assays with the ICC was 0.901 (95% CI 0.885–0.931). At a single cut‐off of CPS ≥ 1, the two assays showed a Cohen’s kappa of 0.891 (CI 0.825–0.957) with an OPA of 98% (CI 95–99%), whereas, at a cut‐off of CPS ≥ 20, the kappa value was 0.808 (CI 0.753–0.862) with an OPA of 90% (CI 87–93%); both of these are in the range of almost perfect agreement. At a cut‐off of CPS ≥ 1, the PPA and the NPA between clones were 98% (CI 95–99%) and 97% (CI 85–100%), respectively. At a cut‐off of CPS ≥ 20, the PPA and the NPA between clones were 95% (CI 90–98%) and 87% (CI 80–91%), respectively. When we considered the three cut‐off categories CPS < 1, a CPS between 1 and <20, and CPS ≥ 20, the weighted kappa was 0.878 (CI 0.813–0.943) with an OPA of 88% (CI 84–92%). The rate of agreement between assays was high at all cut‐offs, and was best for the most relevant cut‐off CPS ≥ 1, whereas the kappa values were always in the range of almost perfect (Table 2).

**Table 2:** Measure of agreement

Measure of agreement	Results
ICC among pathologists, 22C3	0.834 (CI 0.758–0.896)
ICC among pathologists, SP263	0.868 (CI 0.803–0.918)
ICC between clones	0.911 (CI 0.885–0.931)
Kappa at CPS ≥ 1 between clones	0.891 (CI 0.825–0.957)
OPA at CPS ≥ 1 between clones	98% (CI 95–99%)
PPA at CPS ≥ 1 between clones	98% (CI 95–99%)
NPA at CPS ≥ 1 between clones	97% (CI 85–100%)
Kappa at CPS ≥ 20 between clones	0.808 (CI 0.753–0.862)
OPA at CPS ≥ 20 between clones	90% (CI 87–93%)
PPA at CPS ≥ 20 between clones	95% (CI 90–98%)
NPA at CPS ≥ 20 between clones	87% (CI 80–91%)
Kappa for three categories (CPS < 1, CPS ≥ 1, and CPS ≥ 20)	0.878 (CI 0.813–0.943)
OPA for three categories (CPS < 1, CPS ≥ 1, and CPS ≥ 20)	88% (CI 84–92%)

CI, confidence interval; CPS, combined positive score; NPA, negative percentage agreement; OPA, overall percentage agreement; PPA, positive percentage agreement.

## Discussion

The introduction of immunotherapy with anti‐PD‐1/PD‐L1 inhibitors has resulted in remarkable improvements in the outcomes of several advanced solid tumours.^20^ Recently, the FDA approved the use of pembrolizumab as first‐line monotherapy for recurrent and/or metastatic HNSCC in those patients with CPS ≥ 1 evaluated with an FDA‐approved companion test, i.e. the 22C3 PharmDx assay. Since then, the EMA and UK’s NICE have approved pembrolizumab, both as monotherapy and in combination with chemotherapy, as a first‐line treatment for metastatic or unresectable recurrent HNSCC in patients whose tumours express PD‐L1 with CPS ≥ 1, regardless of the test (antibody and IHC platform) used. The registration of an anti‐PD1/PD‐L1 inhibitor for clinical practice is associated with a specific diagnostic assay and staining platform, as well as an immunohistochemical score with specific cut‐off values for patient selection. However, the majority of pathology departments do not have access to more than one of the staining platforms, resulting in an inability to provide full screening for all of the available checkpoint inhibitor drugs in different tumours. The alternative use of LDTs can be limited by the difficulty in standardising many of the assay components. Thus, LDTs are likely to be less robust than commercial tests and may be a source of variability in results. Whenever a companion diagnostic test is not required, comparative studies between different immunohistochemical assays are needed to assess the interchangeability of the different antibodies and platforms at given cut‐off values, as previously reported for some solid tumours such as non‐small‐cell lung cancer.^21^ Moreover, the evaluation of PD‐L1 staining may be affected by its heterogeneous expression within tumour samples and by the interobserver variability.^22^, ^23^ In the last 5 years, only a small number of studies were published dealing with the interpretation of PD‐L1 staining in HNSCC. Moreover, they were based on different evaluation criteria (considering either immune or neoplastic cells, or both) and variable cut‐off values for positivity, affecting the reproducibility of the results. In this study, we aimed to investigate the concordance between the two most common PD‐L1 assays (the 22C3 PharmDx assay and the SP263 assay) on a cohort of HNSCCs. We demonstrated significant similarity in the results of CPS evaluation when we compared the 22C3 PharmDx assay with the SP263 assay (*P* < 0.0001). In addition, we found that the interobserver reliability among pathologists for the continuous scores of CPS with the ICC and the correlation between the two assays were both good. Moreover, at CPS cut‐offs of ≥1 and ≥20, the two assays showed Cohen’s kappa vaues of 0.891 with an OPA of 98% and 0.808 with an OPA of 90%, respectively, both of which in the range of almost perfect agreement. The rate of agreement between assays was high at all cut‐offs and was best for the most relevant CPS cut‐off of ≥1, and the kappa values were always in the range of almost perfect.

Although the study was carried out on a small number of cases, our report presents some substantial novelties. In fact, we restricted our analysis only to two platforms (the Autostainer Link 48 with the EnVision DAB Detection System for the 22C3 PharmDx assay, and the Benchmark XT staining system and the OptiView Universal DAB Detection Kit for the SP263 assay) with the same immunohistochemistry protocol. This approach greatly reduced the variability related to the use of different immunohistochemical platforms and protocols, as recently highlighted by the study of Crosta *et al*., which compared the performance of five different PD‐L1 protocols with the the 22C3 PharmDx assay on 15 cases/30 cores.^18^

A further point of innovation as compared with previous reports is represented by the histological material analysed. Our samples consisted of whole sections from 27 biopsies and 16 surgical specimens of HNSCC. This type of sample represents the patient’s tumour with the full variability in PD‐L1 expression as in the real‐world setting. However, the results of evaluation of PD‐L1 on these samples were more homogeneous than those obtained with TMAs. Indeed, a previous report provided evidence that a single TMA core is not representative of the whole tumour section, with a 0% negative predictive value of single‐negative and double‐negative biopsies being found with a CPS cut‐off of ≥1.^24^ This could explain, at least to some extent, the difference between our results and those of the study of De Ruiter *et al*., which reported precisely on the ICC and Cohen’s kappa for the comparison with CPS values among the reference standard and both the the SP263 assay and an LDT with 22C3 on the Ventana platform.^17^ De Ruiter *et al*. demonstrated that, in a serial section of a TMA containing 147 HNSCCs, the concordance between the 22C3 PharmDx assay and the SP263 assay was lower than moderate with the ICC and in the range of fair (0.20–0.40) with Cohen’s kappa, both at cut‐offs of 1 and 20, with no significant increase being seen at the highest cut‐off, whereas the LDT showed an ICC of at least moderate in both the comparisons and concordance of kappa values from fair to substantial with high variability.^14^ The authors did not insist on tumoral heterogeneity, but concordance investigation among TMA cores and whole sections was carried out in only a subset of 12 tumours of 147.

Finally, we believe that a strength of our study is the number of pathologists who evaluated the expression of PD‐L1 in the HNSCC cohort. In contrast to other reports, in which the number of pathologists involved in PD‐L1 evaluation in HNSCC was small, and the pathologists often did not have specific expertise in the field, this study involved 10 pathologists from 10 different centres who, after taking a training course on the evaluation of the CPS in HNSCC, showed almost perfect agreement, which was best for the most relevant CPS cut‐off of ≥1, and with kappa values always in the range of almost perfect.^17^, ^18^, ^25^ The concordance between pathologists in CPS evaluation was excellent, and implies that, when a pathologist is adequately trained in reading the CPS, the choice of assay does not affect the assessment. This is in line with studies from a Canadian group that investigated the concordance among pathologists with the use of several clones, also in a cohort of HNSCCs, and that showed a high level of agreement for the evaluation of PD‐L1, even with separate evaluation of tumour and immune cells, highlighting how the SP263 clone achieves the best performance in terms of interobserver reproducibility.^25^, ^26^, ^27^

The patients of our cohort were all untreated. Although studies seem to indicate a role for cisplatin‐based therapeutic regimens in the induction of PD‐L1 expression in HNSCC, other studies investigating the effects have provided controversial data.^28^, ^29^, ^30^ Moreover, most recent studies have demonstrated that radiotherapy could deeply affect the tumour microenvironment and the immune response against tumour, also influencing the expression of PD‐L1 (abscopal effect).^31^

Our study had some limitations. First, a low number of cases was analysed, and some specific head and neck areas and metastatic sites were relatively under‐represented. Second, the cases were assessed remotely by pathologists using their own personal workstations. This implies a potential limitation in the standardisation of viewing displays and network bandwidth.

In conclusion, our data demonstrate the substantial interchangeability between the SP263 assay and the 22C3 PharmDx assay for PD‐L1 evaluation in HNSCC patients. Standardised methods (immunohistochemical protocol, antibody, and IHC platform), whole section tissue samples and training could deeply impact on PD‐L1 evaluation and the concordance between different anti‐PD‐L1 antibodies in HNSCC patients. Further studies in other independent cohorts are needed to confirm our data and definitively support the harmonisation of the different PD‐L1 assays.

## Conflicts of interest

The authors declare that they have no conflicts of interest.

## Funding

This study was supported by Università Cattolica del Sacro Cuore, Fondi d’Ateneo, Linea D1 (2018 and 2019; M. Martini).

## Author contributions

B. Cerbelli, I. Girolami, G. D’Amati and M. Martini were the principal authors and the main contributors to writing of the manuscript. B. Cerbelli, L. Costarelli, S. Taccogna, R. Scialpi, M. Benevolo, T. Lucante, P. L. Alò, F. Stella, M. G. Pignataro, G. Fadda and M. Martini analysed and interpreted the data. I. Girolami and A. Eccher performed the statistical analysis. M. Martini and B. Cerbelli performed the immunohistochemical analysis. M. G. Pignataro digitised the slides and uploaded the file on a shared web platform. B. Cerbelli, I. Girolami, A. Eccher, G. D’Amati and M. Martini read and corrected the manuscript. All authors read and approved the final manuscript.

## Ethics approval and consent to participate

All patient data were collected anonymously, and written informed consent, as part of the routine diagnosis and treatment procedures, was obtained from patients or their guardians according to the Declaration of Helsinki. The study adhered to Good Clinical Practice guidelines.

## Data Availability

The data that support the findings of this study are available from the corresponding author upon reasonable request.
